# Extremely Precise Blood–Plasma Separation from Whole Blood on a Centrifugal Microfluidic Disk (Lab-on-a-Disk) Using Separator Gel

**DOI:** 10.3390/diagnostics12112873

**Published:** 2022-11-20

**Authors:** Ali Hatami, Maryam Saadatmand

**Affiliations:** Department of Chemical and Petroleum Engineering, Sharif University of Technology, Azadi Ave., Tehran 11155-9465, Iran

**Keywords:** plasma separation, lab-on-a-disk, centrifugal microfluidic, separator gel

## Abstract

Due to the expansion of point-of-care devices, proposing a convenient and efficient method for blood–plasma separation would help with the use of point-of-care devices. Commercial microfluidic chips are only able to separate a limited amount of plasma, and the majority of these chips need an active valve system, which leads to increase manufacturing cost and complexity. In this research study, we designed a centrifugal microfluidic disk with a passive valve for ultra-accurate and efficient blood–plasma separation on a large scale (2–3 mL). The disk contained a separator gel, which, after applying the centrifugal force, separated the plasma and red blood cells. The passive valve worked based on the inertial force and was able to transfer more than 90% of the separated plasma to the next chamber. The results demonstrated that the separated plasma was 99.992% pure. This study compared the efficiency of the disk containing separating gel with the common lab-on-a-disk design for plasma separation. A comparison of the results showed that although the common lab-on-a-disk design could separate almost pure plasma as the disk contained separator gel, it could only transfer 60% of plasma to the next chamber.

## 1. Introduction

Plasma and serum are two common laboratory samples that are extracted from human blood using centrifugal force. Plasma contains 92% water, 7% proteins, 1% fat, hormones, vitamins, cell-free DNAs, exosomes, etc. [1]. Considering that nowadays, due to the high potential of microfluidic devices, the use of them is on the rise, proposing an appropriate method for plasma isolation on a micro-scale platform is very important. Although the blood–plasma separation is easily accomplished through laboratory methods (huge centrifugal system) with the help of an operator, it is more challenging on the small-scale system without an operator. Centrifugal microfluidic devices, also known as lab on a disk (LoaD), are one of the microfluidic systems with the highest potential for clinical applications such as DNA and cfDNA extraction for early disease diagnosis [2,3]. As mentioned earlier, developing plasma separation on a microfluidic device such as LoaD is fraught with challenges, one of which is the transfer of isolated plasma to a new chamber for the rest of the process, which can happen through an active or a passive valve [4]. Although utilizing an active valve for this purpose is easier, it requires the fabrication of a more complex valve that results in a more expensive device. As a result, usually, passive valves achieve greater simplicity and lower manufacturing costs. In 2016, Kim et al. proposed a reversible valve for the centrifugal microfluidic disks [5]. The valve was operated based on push and twist and was controlled by a computer system. Although the use of this valve led to better control of the sample displacement, each time the disk had to be be stopped in order to activate or deactivate the valve, and the disk fabrication was more complicated and expensive. Passive valves, such as siphon valves, operate based on inertial force, which occurs when the rotational speed of the disk suddenly decreases or stops. In 2010, Amasia et al. designed a centrifugal microfluidic disk for large volume blood–plasma separation. The designed disk was capable of extracting plasma from 2 mL of blood [6]. In order to transfer the separated plasma to the next chamber, the disk had a passive siphon valve. One of the main challenges of using inertial-based valves is the inadvertent re-mixing of red blood cells (RBCs) and plasma after a sudden reduction or cessation of the disk-rotation speed. In 2015, Kua et al. designed a centrifugal microfluidic disk for blood–plasma separation. In this disk, neither passive nor active valves were used. In order to separate plasma, a curved microchannel was designed. When the blood, based on the centrifugal force, flowed in the microchannel, RBCs, due to their density, tend to approach the outer radius of the curve. At the end of the microchannel, the outer radius and the inner radius were divided into two microchannels. Therefore, the RBCs were separated from the plasma [7]. In 2019, Hu et al. used COMSOL software to optimize a funnel shape chamber for blood–plasma separation from a large volume of blood. By reducing the cross-sectional area of plasma and RBCs, the possibility of re-mixing decreased after applying the inertial force. However, in order to err on the side of caution, the siphon valve was designed in a way that it could not extract all of the separated plasma [8]. As a result, there was always a small amount of plasma left in the chamber. In 2021, Shi et al. designed a centrifugal microfluidic disk. The disk possessed multi chambers and multi microchannels such that the plasma chamber and blood chamber were collinear with the center of the disk [9]. It is important to mention that since the concentration of some components in the blood plasma is so low, such as cfDNA, maximum plasma separation is usually required. Therefore, researchers have been working on microfluidic devices to address this issue [9,10,11,12].

In this study, we propose a novel method for maximizing pure plasma separation from a large volume of blood using a centrifugal microfluidic disk. To the best of our knowledge, this is the first application of a separating gel in the centrifugal microfluidic system that not only has the potential to be used for plasma separation but also can be used for other purposes such as valving systems. Finally, this study evaluates the efficiency of the disk containing separating gel through comparison with the common lab-on-a-disk design for plasma separation.

## 2. Materials and Methods

In this research, two centrifugal microfluidic devices were fabricated for blood–plasma separation. The first disk contained a separator gel and it was called Gel-Disk. The second disk, analogous to common laboratory disks, was designed to separate blood plasma, called NoGel-Disk. It is worth mentioning that all the experiments in this research were repeated three times, and to measure the separated plasma an insulin syringe (Nikan Company, Tehran, Iran) was used. To analyze the purity of the separated plasma, hemocytometry as well as a hemoglobin assay kit (Zist Shimi Inc., Tehran, Iran) were utilized. The technique for using the hemoglobin kit was described in our previous research [13]. For this purpose, at the first, a wide range of hemoglobin concentrations were prepared; then, their absorbances were measured with a UV–VIS spectrometer machine (Hach, Loveland, CO, USA) and a calibration graph was drawn. Then, the absorbance of the samples was measured and compared to the calibration graph. In order to measure the number of white blood cells (WBCs) in the sample, the hemocytometry method was utilized. For this purpose, at first, samples were diluted, and then they were put on a hemocytometer (Micron, Shanghai, China) and cells were counted under an optical microscope (United Scope LLC, Irvine, CA, USA). A 10× objective lens was used to clearly distinguish between the cells. Finally, based on the following formula, the number of cells was reported.

(1) $$\mathrm{Cells}/\mu L=\frac{\left(\mathrm{number}\;\mathrm{of}\;\mathrm{cells}\;\mathrm{counted}\right)\left(\mathrm{dilution}\;\mathrm{factor}\right)}{\mathrm{number}\;\mathrm{of}\;\mathrm{large}\;\mathrm{squres}\;\mathrm{counted}}\times 10^{7}$$

### 2.1. Instruments

A rotating platform was utilized to exert centrifugal force on the disk. The rotating system was already designed and fabricated in our research group and was able to rotate clockwise as well as counterclockwise up to 7000 g [14]. In order to control the acceleration and deceleration speed of the disk, a software program was designed and connected to the rotating system. The rotating system included a stroboscope that could be used to easily observe the disk while still in motion [13].

### 2.2. Gel-Disk Design

**Figure 1:**
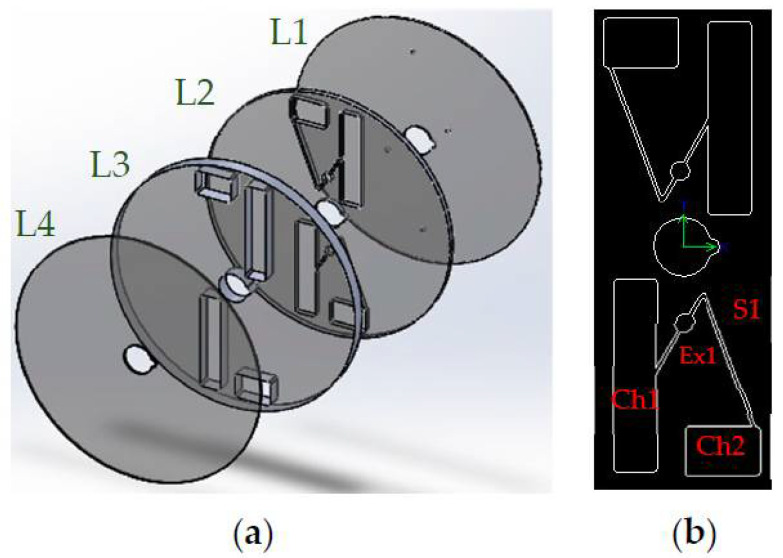
Schematics of the layers of the Gel-Disk. (**a**) Disk PMMA layer order. (**b**) Schematic of blood chambers and passive siphon valves.

The disk was made of four poly methyl methacrylate layers (PMMA, Chochen, Tainan, Taiwan); the first layer (L1) was 0.5 mm and was designed for holes and vents. The second layer (L2) was 1 mm and was designed for siphon valves. The third layer (L3) with a thickness of 6 mm was designed for chambers. The capacities of the blood chamber (Ch1) and the plasma chamber (Ch2) were 4 mL and 2.5 mL, respectively. The fourth layer (L4), called the bottom layer, possessed a thickness of 0.5 mm. It is worth mentioning that Ch1 and Ch2 were connected to each other by using a siphon valve (S1). The width of S1 was 1 mm, and it had an expander with a radius of 1.5 mm to prevent passing blood from the peak of the siphon at the initial time. Figure 1 presents schematic arrangements of the Gel-Disk layers and the designed geometry. In this disk, two sets of separation chambers were designed, both of which were identical. The diameter of the disk was 13 cm and was designed for a 3 mL sample of blood with around 45% hematocrit. To design microchambers, microchannels, valves, etc., SolidWorks 2014 software was used, and all the patterns were cut using a laser-cutting machine (CO_2_ Laser Machine 6090, Beijing, China) on the PMMA layers. Chloroform, which was used in the literature to adhere the layers together [15], welds PMMA immediately; therefore, it was important to align all the layers properly. Diluted chloroform (Sigma-Aldrich, Taufkirchen, Germany) was used to fabricate this disk due to the need for a fairly high centrifugal force to displace the gel through the blood chamber. If double-sided adhesive tape had been used, the blood chamber might have leaked due to the high centrifugal force. However, no leakage was observed when the layers were bonded together by diluted chloroform. It should be noted that an expander (Ex) was considered in all of the designs to prevent the injected blood from passing through the siphon peak at the initial time, Figure 1b [16].

### 2.3. NoGel-Disk Design

**Figure 2:**
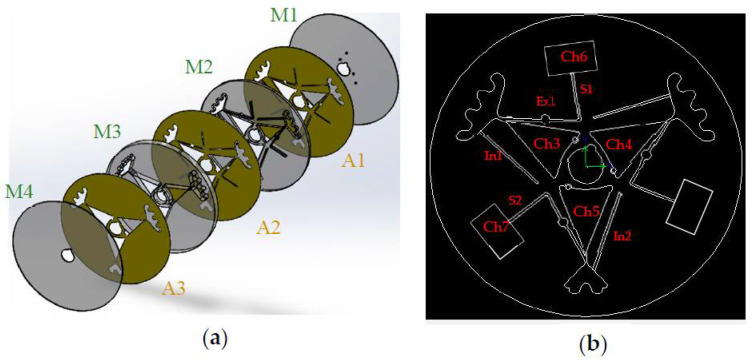
Schematics of the NoGel-Disk layers. (**a**) Disk layer order including PMMA (M) and double-sided adhesive (A). (**b**) Schematic of blood chambers and passive siphon valves.

Like the Gel-Disk, the NoGel-Disk was made of four PMMA layers (Chochen, Tainan, Taiwan) containing four layers with different thicknesses. The first layer (M1) was 0.5 mm and was designed for holes and vents. The second layer (M2) was 1 mm, and all the siphons and expanders were designed on this layer. The third (M3) and fourth (M4) layers with thicknesses of 6 mm and 0.5 mm were designed for the chambers and the bottom of the disk, respectively. The diameter of the disk was 13 cm and was designed for 3 mL of blood with around 45% hematocrit. All of the layers were adhered together with a double-sided adhesive (Tesa, Norderstedt, Germany) tape with a thickness of 65 µm. Before cutting the pattern, one side of the first adhesive layer (A1) and the second adhesive layer (A2) were taped to the top and bottom of M2, respectively. Additionally, one side of the third adhesive layer (A3) was taped to the bottom of M3. After that, the patterns were cut using a laser-cutting machine (CO_2_ Laser Machine 6090, Beijing, China). The order of adhesive and PMMA layers prevented the adhesive layers from contacting the blood samples. Figure 2 illustrates the order of the Gel-Disk layers and the designed geometry. Finally, all of the disk layers were aligned and sandwiched under a manual screw press machine (which was fabricated in the research group, Iran) for 1 day. In the NoGel-Disk, 3 sets of separation chambers (Ch3, Ch4, and Ch5) were arranged on the disk. Ch3 and Ch4 were designed identically to separate plasma from 3 mL blood, and Ch5 was designed for 2 mL of blood. Each siphon valve had an expander to prevent passing blood from the peak at the initial time. The cross-sectional areas at the narrow point of the plasma separation chamber for all the designs were 1 × 7 mm^2^. For each blood chamber (Ch3, Ch4, and Ch5), one inlet channel was designed (In1, In2). The purpose of these inlet channels was to facilitate blood injection. The operator not only could use the vent that was designed on M1 but also was able to use these inlet channels. The width of inlet channels was 1 mm, and after blood injection they were sealed with a tape.

### 2.4. Separator Gel

Centrifugal force is one of the most important factors in designing lab-on-disks. One of the applications of this force is particle separation. The centrifugal force leads to the sedimentation of red and white blood cells in the base of vials. The RBC, WBC, and human blood plasma densities are approximately 1.11 g/mL, 1.08 g/mL, and 1.025 g/mL, respectively [17]. By utilizing a biocompatible gel with a density between 1.08 g/mL and 1.025 g/mL [18], after applying centrifugal force the separator gel would be located between the buffy coat and plasma, which would prevent remixing. The gel was extracted from the commercial blood-plasma tubes (Vacutainer^®^ PST™ Tubes) and placed in the Ch1 of the Gel-Disk during the fabrication. In order to extract the gel, one blood-plasma tube was cut, and the gel was extracted by a stainless steel spatula (IMS, Shanghai, China). It is important to mention that the volume of gel was 0.5 dm^3^.

### 2.5. Clinical Sample

Blood samples were collected from volunteers, who were aware of the purpose of the research, at Sharif University Medical Clinic under the supervision of a specialist. The blood samples were stored in the heparin blood tubes (Vacutainer^®^) and tested by the disk on the same day. Before testing, the blood hematocrit of the samples was noted to be 45 ± 0.5 percent.

## 3. Results and Discussion

### 3.1. Gel Displacement

**Figure 3:**
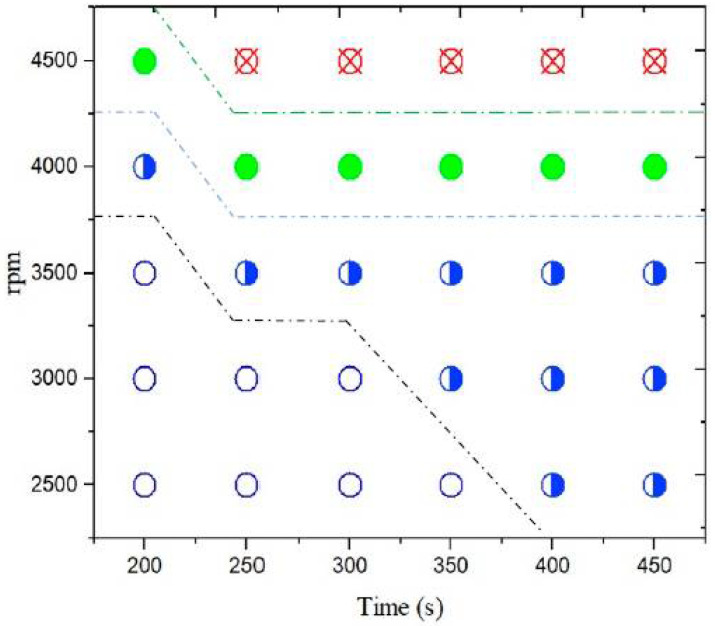
The relationship between CF force, time, and gel displacement. The blank circles, half-filled blue circles, full green circles, and red cross circles represent no plasma separation/no gel displacement, plasma separation/no gel displacement, plasma separation/gel displacement, and erythrocyte rupture, respectively.

Usually, the centrifugal force (CF) that is used for plasma separation is between 500 to 2000 g force [19]. As both PMMA and the separator gel are hydrophobic, the gel tends to stick to the PMMA [20], and a fairly high centrifugal force was required for gel displacement. On the other hand, the centrifugal force had to be chosen such that the RBCs cells would not rupture during the separation process. Therefore, finding an appropriate centrifugal force for displacing the gel and not rupturing the RBCs in the Gel-Disk was important. The gel started to move at 2300 g (4000 rpm, r = 13 cm) and after 250 s was located precisely between the plasma and the buffy coat. Figure 3 demonstrates the relationship between the centrifugal force, time, and their effects on the gel displacement, and erythrocyte rupture. For example, after 250 s from starting the rotating profile, at 2500 and 3000 rpm not only did the gel did not move but no pure plasma separation occurred either. At 3500 rpm, only the plasma was separated, and the gel did not move. However, at 4000 rpm, both plasma separation and gel displacement occurred. A further centrifugal force led to the RBCs’ rupture.

### 3.2. Speed Protocol of Plasma Separation

**Figure 4:**
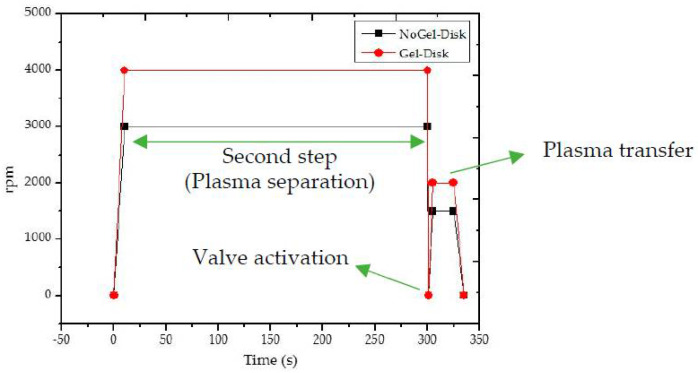
Speed protocol of the Gel-Disk as well as NoGel-Disk.

The plasma separation steps for the Gel-Disk as well as NoGel-Disk were identical (Figure 4). Both Gel-Disk and NoGel-Disk had a rapid initial acceleration to 4000 and 3000 rpm in 10 s, respectively. Then, the separation step, as the second step, lasted 300 s. The third step was the activation of the valve, which occurred with a sudden stop of the disk in one second and then rapid acceleration to 2000 rpm and 1500 rpm for Gel-disk and NoGel-Disk, respectively. In the final step, the disk continued to rotate at the given speed for 10 s to transfer the plasma to the next chamber and then gradually slowed down to stop completely in 10 s. Figure 4 shows the speed protocol of the both disks. All rotations were clockwise.

### 3.3. Blood Plasma Analysis of Gel-Disk

**Figure 5:**
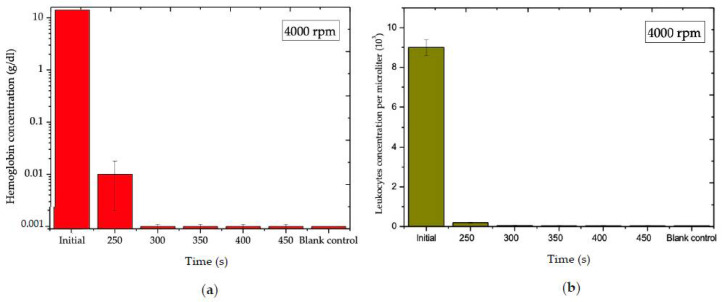
(**a**) Effect of time on the hemoglobin concentration at 4000 rpm for Gel-Disk. (**b**) Effect of time on the white blood cells concentration for Gel-Disk.

**Figure 6:**
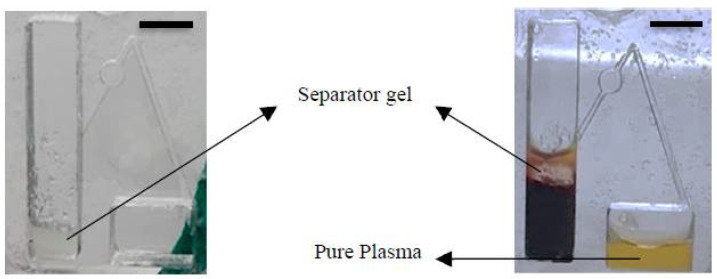
Photograph of Gel-Disk at the initial and at the end of plasma separation. Scale bar is 1 cm.

As mentioned before, the optimum rotating rate for plasma separation with gel displacement occurred at 4000 rpm. Therefore, an analysis of the effect of time vs. hemoglobin concentration at 4000 rpm was required. In this research, the quality as well as the quantity of the separated plasma were analyzed. The results demonstrate that the hemoglobin concentrations of the blood samples were 0.001 ± 0.0004 g/dL, equal to roughly 99.992% purity of the plasma at 300 s, 4000 rpm. Figure 5a,b show the effect of time on hemoglobin and leukocyte concentrations at 4000 rpm, respectively. By utilizing inertial force and a siphon valve, almost 90 ± 0.4% of the separated plasma was transferred to the next chamber. Figure 6 shows the position of the separator gel initially and at the end of the separation process.

### 3.4. Blood Plasma Analysis of the NoGel-Disk

#### 3.4.1. Blood Plasma Analysis for Ch3 and Ch4

**Figure 7:**
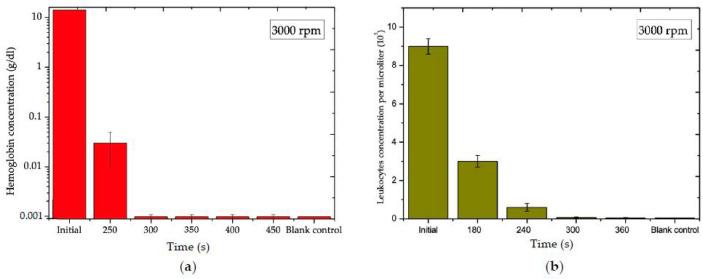
(**a**) Effect of time on the hemoglobin concentration at 3000 rpm for NoGel-Disk. (**b**) Effect on time on the white blood cells concentration for NoGel-Disk.

**Figure 8:**
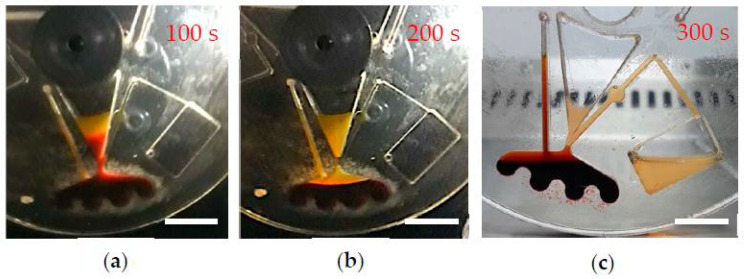
(**a**,**b**,**c**) show images of the plasma separation from 3 mL of blood on NoGel-Disk at 100, 200, and 300 s respectively. Scale bar is 1 cm.

The NoGel-Disk design was able to separate 1 ± 0.04 mL plasma from 3 mL of blood in 300 s, which was equal to approximately 60.6 ± 3.5% of the plasma volume with a purity of 90 ± 0.6%. Although the NoGel-Disk was capable of extracting plasma with comparable purity to the Gel-Disk, the amount of separated plasma compared to the whole plasma was very low due to the prevention of remixing RBCs into the plasma. Figure 7a,b show the effect of time on hemoglobin and leukocyte concentrations at 3000 rpm, respectively. Figure 8 shows plasma separation from 3 mL blood at different times: 100, 200, and 300 s after starting the rotation. It is obvious that 100 s was insufficient for plasma separation; on the other hand, although in 200 s the separation line between plasma and RBCs was observed, the hemoglobin of plasma was still high. It is worth mentioning that a very small amount of blood could penetrate between the disk layers due to the centrifugal force at the end of the separation process. However, since the leakage was very minor, this did not affect the separation process (Figure 8c).

Although both disks were able to reach acceptable concentrations of hemoglobin and leukocytes at 300 s, it is crystal clear that the function of Gel-Disk in transferring the quantity of plasma was not only better than NoGel-Disk, but also the quality of the separated plasma at different times (less than 300 s) was higher, which meant that the separation rate of the Gel-Disk was higher than the NoGel-Disk at any time less than 300 s; however, both disks needed 300 s to reach the highest purity that was mentioned before. For instance, at 250 s the hemoglobin concentration of the plasma separated by Gel-Disk was lower that 0.01 g/dL; however, for NoGel-Disk this amount was almost 0.03 g/dL.

#### 3.4.2. Blood Plasma Analysis for Ch5

The NoGel-Disk design was able to separate 0.7 ± 0.05 mL plasma from 2 mL of blood in 300 s, which was equal to approximately 63.5 ± 2.5% of the plasma volume with a purity of 90 ± 0.6%. The results indicate that all of the blood chamber designs of the NoGel-Disk were able to separate almost pure plasma as the Gel-Disk; however, their efficiency for transferring the quantity of plasma to the next chamber by a siphon valve was lower.

## 4. Conclusions

The separator gel possesses high potential to be utilized in the centrifugal microfluidic (lab-on-a-disk) system. In this research, we were not only able to separate plasma in a completely pure manner (99.992%), but we could transfer more than 90% of the separated plasma to the next chamber by using a very basic passive siphon valve. In addition, we showed that although the widespread design (NoGel-Disk) with the contracted cross-sectional area between the plasma and the RBCs was able to separate almost pure plasma (90 ± 0.6%), it was only able to transfer approximately 60% of the separated plasma to the next chamber.
